# The Port-Folio; or, Medical and Surgical Miscellany

**Published:** 1819-04-01

**Authors:** 


### FOURTH DEPARTMENT.

THE

### Port-Folio;

OR,

### MEDICAL AND SURGICAL MISCELLANY.

### I.

*To the Editor of the Medico-Chirurgical Journal.*

SIR,

THE laudable professional spirit, which has induced Dr. Porter to publish so candidly his opinions and cases of hydrencephalus,* will incline him also to receive with equal candour the remarks I take the liberty to subjoin on his valuable communication in your excellent Journal published this month. That a disease will arise in the brain producing effusion and constituting hydrencephalus, strictly so called, unconnected with any previous disease of another organ, there can be I think little or no doubt. I was, therefore, very much pleased with the ingenious idea Dr. Porter has thrown out, and with the discrimination he has made respecting the difference of the absorption of the effused fluid, when produced by inflammation of the substance of the brain and of its membranes. I was much gratified by the anatomical appearances he has described, the result of inflammation in the posterior part of the brain, with his excellent observations upon them as connected with hydrencephalus; they at once mark a mind zealous and acute in the pursuit of its object. But, Mr. Editor, I request permission to call Dr. Porter's attention to the condition of the chylopoietic organs and intestinal functions previous to that morbid state of the brain which terminates in hydrencephalus. About four years have now elapsed since my letter to Dr. Wall was published, in which I called the attention of the public to this mode of considering hydrencephalic affections of the brain. During this period my attention has been closely given to this subject; and if my former experience and conviction had wanted confirmation, they would have amply received it from the additional facts which have since presented themselves to my observation. The doctrine of sympa-

* I continue to use this term as more appropriate than hydrocephalus, but both are improper, and are not more expressive of the nature of the disease than hydrothorax is of pneumonia or pleuritis, when watery effusion is the result of these diseases.

thetic action in the production of disease, has of late been very scientifically examined, and the attention that has been paid to the consequences which deranged digestive organs produce, sympathetically considered, in generating distant morbid action, has given rise to much improvement in the practice of physic. Previous to this mode of considering diseases, every part was treated almost as an insulated organ, without any reference to its connection, either partially or generally, with the whole system; hence arose the specific remedies which were supposed to have an individual effect upon each separate organ. But, whoever for a moment views the body anatomically, and reflects upon the vital principle, must at once see what important effects may be, and are, produced by the disease of almost any one organ, on various other parts, which apparently have no connection. Organs which readily sympathise at one period of life, are not so readily thrown into such action at another; this may probably be explained from the growth of the body during the progress to maturity giving particular determinations of blood, and from the change in the balance of the circulation when we are descending into the vale of years. This sympathy will arise, either from nervous irritation, or from obstruction to the flow of blood to a particular part, thus throwing a greater quantity elsewhere; and so intimately connected are the nerves and blood-vessels in all their minute ramifications, and so dependent upon each other in their functions, that one cannot be affected without the other being thrown into morbid agitation: hence the sensorium, the origin of the one system, and the heart, by means of which the other mainly transmits its contents, must suffer and produce corresponding effects upon the general system: and convulsions and fever are no uncommon result.

To a gentleman of Dr. Porter's reflection, the parts of the body which have been most frequently observed to sympathise with each other, need not be pointed out. The obvious one, the subject of present consideration, is between the digestive organs and the brain. The ready affection of the brain, in very young people, from irritation of the digestive organs, may in fact receive its illustration from the greater quantity of blood carried thither during its growth, for the full developement of the sensorium. The rapidity with which morbid action is not uncommonly transmitted from one part of the body to another is very remarkable, and is often such, that there shall be no trace whatever in the first link of the chain when the ultimate one shall prove fatal, and be sufficiently visible upon dissection after death. Dr. Porter will readily call to mind the retrocession of rheumatism to the heart, gout to the stomach, and of erysipelas to the brain: no mark of these diseases shall appear after retrocession, although sufficiently apparent and severe before it, when the brain, the heart, and the stomach shall be found diseased upon dissection, but shall have exhibited no symptom of disease until such retrocession took place. This observation is equally applicable to the connection between the irritation of the digestive organs and the production of hydrencephalus, although the brain shall be found solely in possession of extensive diseases after death. The reciprocal sympathy, which exists between the head and abdominal viscera is such, that neither can be long much affected without some part of the other being called into morbid action. Unquestionably, when there is either a conversion of disease from the digestive organs to the brain, or a synchronous morbid action existing between them, the latter should be an object of watchful practice,* without, however, neglecting to pay particular attention to the former. From considering hydrencephalus as a disease of the head only, without regarding the state of the abdominal viscera, has arisen the fatality which formerly almost uniformly attended it, of which Dr. Porter can very soon satisfy himself by reference to authors.† It is not enough to purge the bowels and occasionally to keep them open: this will not answer the purpose of a safe curative indication. The peristaltic motion of the intestines is torpid and irregular, they must be kept in gradual movement, so that no part of them be suffered to be quiescent.‡

The valuable observation which Dr. Porter has made of "avoiding all unnecessary irritation from much calomel and other drastic purgatives," I have found particularly applicable to some apoplectic attacks arising from irritation of the chylopoietic viscera. It is but justice to the late Dr. Kirkland, of Ashby, to say, that the avoidance of the harsher treatment was first, as far as I know, suggested by him in his very excellent Commentary on Apoplectic and Paralytic Affections; a work replete with much useful practical knowledge. He strongly recommends the pulvis antimonialis; and it would seem, that the Irish physicians of reputation,§ as well as my own experience, have confirmed Dr. Kirkland's recommendation.

The general principles upon which the view I have taken of the subject of hydrencephalus is founded, are stated in my Letter to Dr. Wall, and there is now in the press an Appendix to that Letter, containing a detail of cases, with practical illustrations of the doctrines which I entertained, not only of the connection between a derangement of the digestive organs and the brain, but also of the effects which irregular intestinal actions have in deranging the chylopoietic viscera in producing that morbid condition of the encephalon which ends in water of the brain. I need not, therefore, at this time, extend my observations, nor enter into minute particulars; but I beg to repeat in conclusion, that I by no means intend to deny that a diseased action does occur, not unfrequently, in the brain, per se, independent of previous disease in

---

* *See* p. 61-66 of the Letter to Dr. Wall.

† *See* Note, p. 7, of the Letter, and also p. 49.

‡ *See* p. 37 of the Letter to Dr. Wall.

§ Of the Virtues of James's Powder in Apoplectic Diathesis, by J. Cheyne, M.D.; Dublin Hospital Reports, Vol. I. p. 315.

any other organ. I repeat this, because I appear formerly to have been misunderstood; experience has, however, seriously impressed upon my mind the close connection which exists between a deranged action of the digestive organs and intestines, and the production of hydrencephalus.

I am, &c.

Your very obedient servant,

Queen Street, May Fair, G. D. YEATS.

January 7, 1819.

---

### II.

### *Dr. M'Whirter's Account of a Surgical Operation.*

( Communicated by the Army Medical Board. )

I HAVE lately read the account of M. Richerand's Operation on the Chest, and the Commentaries of M. M. Deschamps and Percy on the same. I do not conceive that this solitary case of daring surgery, will afford a sufficient basis for the anticipations of National superiority, which the two latter Gentlemen seem inclined to indulge. Nor do I allude to the ultimately unsuccessful termination of M. Richerand's operation, but because I am confident that many equally bold exploits have been performed by British surgeons, though they have not been recorded with that pomp and eagerness with which every surgical event is registered in France. On this account I deem it a duty to departed merit, to rescue from oblivion the following imperfect account of an operation, at least as bold, and more successful than M. Richerand's, performed by a gentleman, now dead, but at that time surgeon to His Britannic Majesty's forces.

During one of the battles of the Helder campaign, Lieutenant Wynne, of the 2d Battalion, 17th Infantry, to which I was attached, was brought to me from the field, as I thought mortally wounded by a grape shot, which had entered the left side of the chest, and had lodged beyond the reach of my instrument, in its cavity. He was removed from the field with the other wounded, and subsequently conveyed to England, where, to my utter astonishment, I met him afterwards, not only alive, but apparently in perfect health. It may easily be conceived, that I made particular enquiry relative to his almost miraculous recovery, and the following is an outline of his report, every iota of which I as firmly believe as I do my own existence.

He stated that, having lingered several months, with no other expectation than that of death, he was placed under the care of staff surgeon Hunter, then at Harwich, who having cut out portions of two or three of the ribs, extracted a three ounce iron grape shot, together with part of his dress from a depth of five inches in the lungs. I saw the wound, and most solemnly believe the statement of the officer.

In the course of the late war, indeed, both by sea and land, innumerable instances have occurred, evincing the superiority of our medical and surgical, as well as of our naval and military chiefs ver those of France.

F. M'Whirter, M. D.
Member of the Royal College of Physicians of London; and Physician to the Dispensary, Lying-in Hospital, and Fever Hospital, of Newcastle upon Tyne.

---

### III.

### *Cases of Erysipelas, demonstrating its occasionally Contagious Character.*

A young lady, expectorating under phthisis, struck the skin off a small space near to the left elbow, about the 20th of September last. It soon became painful, and was poulticed. The pain increased, and an erythematous affection of the skin extended itself up the arm. On the 28th the family surgeon was sent for. He found an abscess about two inches above the elbow, and two smaller ones at the same distance below it; the arm was nearly covered with a rose coloured efflorescence. She would not permit the abscesses to be opened—leeches were applied, and the poultice was continued. 29th. The abscesses were opened, and the inflammation being considerable, leeches were applied again. On the 30th and each succeeding day, for a week, leeches were applied to some part of the arm, which in many places presented hard nobs, in which she felt the phlegmonous throbbing: during this period the arm was wrapped in old linen wetted with liquor ammoniæ acetatis, properly diluted. Notwithstanding these means, several smal abscesses formed; one of which was opened on the 6th of October, and another on the 9th. Abscesses continued to form in the course of the principal lymphatics from the elbow to the axilla, to the 27th of the month. The Reporter was then called in, on account of the mother of this young lady, who became the subject of the same disease; and a sketch of whose case will be subjoined. To the young lady's arm he ordered a firm roller with oxycrate; and prescribed five grains of carbonat of ammonia every fourth hour in a cup of sarsaparilla decoction. The openings made into the abscesses remained; they were small and sinuous; a faint erythema overspread the arm, with œdema of the hand. Under the pressure of the roller these appearances soon went off. An opening under the axilla was enlarged by the bistoury; and in ten or twelve days, her recovery from the affection of the arm might be deemed complete.

Mrs. ———, the mother, slept with the subject of the above case; on the 10th of October, she felt pain at the point of the middle finger of her right hand. She does not believe the cuticle was abraded. On the next day the inflammation extended upwards to the bend of the arm, in a narrow line, not exceeding in breadth, the sixth or fourth part of an inch. On the 12th, the inflammation at the point of the finger had disappeared; but an erysipelatous blush spread over the back of the hand, and diverged from the line of the inflamed lymphatic up to the elbow; the lymphatic was dark, tense, and knotty. Many leeches were applied, and the discutient lotion, but suppurations took place. The Reporter of these cases saw the patient for the first time on the 27th. The pulse was rather frequent, but neither hard, nor strong; she had headache, and rigor. She was treated with purgatives; one bleeding was prescribed; frequent scarifications were made in the arm; the blood exhibited a gelatinous covering; the scarifications generally educed from one to two ounces of blood, attended by great relief, and healed readily and kindly. On the 13th of November, a large abscess formed near to the insertion of the deltoid muscle, and was opened. Rollers were firmly applied, wetted with oxycrate; the arm gradually recovered its natural condition, excepting a cordy feel in the lymphatic from the finger to the site of the last abscess, for which camphorated oil was ordered; and the Reporter took his leave on the 19th instant.

It is remarkable, that in the daughter the disease was in the left arm; in the mother, it appeared in the right hand; points that necessarily came in contact during sleep, as they occupied only one bed. These, and other cases on record, should induce medical gentlemen to declare the contagious nature of this disease when they meet with it, and to recommend as little contact as possible.

The two following examples of severe and well marked erysipelas of the face proving contagious, occurred to an emminent practitioner;—their authenticity may be depended upon.

I. Mr. C. after using great exertions in throwing water, in which he was standing, to extinguish a fire that had broken out upon his premises, was attacked, in a few days, with erysipelas on one side of the face, which soon spread to the other, and the whole head became enormously swollen, with so considerable a degree of fever and delirium as to demand the abstraction of blood. Vesications followed, and the disease run its usual course. As it began to decline, his lady, who had nursed him, and occasionally laid upon his bed during his illness, was attacked with the same disease precisely, attended with a great degree of fever, and very violent delirium.

II. Eight or ten months after the occurrence of the preceding cases, a gardener, occupying a small house in the garden of a gentleman, was seized with erysipelas of the face, with its usual characteristic symptoms. The same practitioner, after visiting him, was asked by the gentleman if the disease was infectious, that he might prevent all communication between the gardener and the family. He answered that it was not, but immediately recollecting the above instance, added, "as out of nothing comes nothing, I would advise you not to allow the children, or any of the family, excepting one person, to go into the house." The butler was in consequence directed to carry to him whatever he wanted, and orders were given that no other member of the family should visit the sick man. Exactly as in the preceding instance, as soon as the gardener began to recover, the butler was attacked with erysipelas.

In former times, erysipelas was known to become epidemic in the air of an hospital, but this has not occurred of late years, since a better system of ventilation and purification has been adopted. Dr. Bateman observes, "it has been the subject of some discussion whether erysipelas is not sometimes propagated by contagion. The disease has been noticed in several hospitals to prevail in certain wards, among patients admitted with different complaints; but has seldom been known to spread in private houses. Dr. Wells, indeed, has collected several examples (see Transactions of a Society for the Improvement of Medical and Chirurgical knowledge, vol. ii.) of the apparent communication of erysipelas by contagion, which occurred in private families. But such cases are, at all events, extremely rare, and perhaps never happened in well ventilated and cleanly houses." *On Cutaneous Diseases, p.* 130.

The above examples, however, disprove the concluding remark, for they occurred in gentlemens' families, in pure air, and in one instance, where the original disease was evidently artificially induced.

---

### EXCERPTA EPISTOLARIA.

1. *Mr. Grabham's Case of Monstrosity.* Mr. G. met with a curious case of lusus naturæ lately in a young pig. The cranium, in comparison with the face, was proportionally as large as the human; the facial angle very nearly a right angle. From the forehead, which was large, issued a proboscis about an inch and a half in length, and a sixth of an inch in diameter. Its superior half was formed internally of bone; its termination was by a single opening, which seemed to possess a sphincter muscle. Immediately under the proboscis, and near the centre of the face, was a single eye, large and rather prominent. Half an inch below the eye, at the margin of the upper lip, there was a protuberance resembling a nipple. It was not certain whether it had breathed, as it was not discovered till several hours after its birth.

2. *Case of Aneurism.* A middle aged man died suddenly, without any previous indisposition, or suspicion of disease. On opening the body, an aneurism was discovered of the arch of the aorta, which had formed an adhesion with, and burst into, the right auricle of the heart. The consequence was instant death.

3. *Oil of Turpentine in Sciatica.* M. Recamier, a physician in Paris, has lately published a Thesis containing the results of his experience with the oil of turpentine in femoro-popliteal Neuralgia, or, in plainer language, Sciatica. The mode of administration was this: Two drachms of the essential oil were mixed with four ounces of honey of roses, and a third part taken thrice a day. In the space of six days ten were cured out of twenty, and most of the remainder were relieved.

4. Dr. MOULSON, of Halifax, Yorkshire, writes as follows. Typhus is not so prevalent here as it has been. Scarlatina has supplanted it. Cynanche tonsillaris shews itself here and there. Treatment: Puncture the tonsils immediately, and if matter does not flow, a little blood will; a blister round the throat, and a cathartic sets the patient on his legs in a day or too; and seldom do we find any thing more necessary, unless the patient has been ill some time before we are called in, and then the decoct. cinchonæ c. acid. muriat. after the abovementioned treatment soon sets him on his legs again.

5. Mr. BUSHEL about two years ago, prepared a tincture of kino according to the College formula, which was powerfully astringent, and of a good colour, &c. About twelve months after it was made, it began to get thick, and in six months more, it had become converted into a substance resembling *gelatin*, and the astringent taste nearly gone. This change could not have taken place in consequence of evaporation, or exposure to the air, for the bottle was well stopped, &c. It is insoluble in æther, alcohol, proof spirit, or water.

6. *Extirpation of the Uterus.* In addition to those already on record, we have lately learnt the following instances. A poor woman, nearly worn out by hæmorrhage and irritation, from inversio uteri, had the ligature applied by Mr. Windsor, under the sanction of Dr. Hull, of Manchester, on the 22d of last August. The uterus was separated on the thirteenth day from the application, and the patient, we believe, did well. A case of chronic inversion of the uterus has lately come under the care of a surgeon at Altringham, near Manchester, where we have reason to believe the ligature has also been successfully applied. A case has been well authenticated within these few months, where the uterus sloughed entirely away, and the patient did well.

7. *Accidents from drinking cold Water when the Body is heated.* It has been long known, that accidents from this cause are always alarming, and frequently fatal. The late celebrated Dr. Currie advised a large opiate as the best remedy. A physician in America, praises the superior efficacy of strong tinct. camphoræ. If we can trust the accuracy of our information, this physician, when lately called to a case of this nature, dissolved half an ounceof camphor, in half a pint of brandy, and gave one third of this solution every ten minutes, with the best effects.

---

The subject of the Plague and Quarantine is now before parliament. Dr. James Johnson, the Editor of this Journal, underwent a long examination on the 17th of March, before a select Committee of the House of Commons, relative to Contagion, Endemic and Epidemic Diseases. The wild reveries, lately broached on these subjects are now meeting what they deserve—a complete exposure and refutation.

J. Hennen, Esq. Inspector of Military Hospitals, Dr. Muttlebury, Dr. Marshall Hall, and Captain Scoresby, have been elected Fellows of the Royal Society of Edinburgh.

Dr. J. B. Davis, senior physician of the London Dispensary; senior physician of the Universal Dispensary for Children; and physician of the Surrey Dispensary; will commence his Lectures on that branch of the practice of medicine which relates to the diseases, and medicinal management of children and young persons, at the above Institution, on Thursday the 1st of April. In this course, an extensive view will be taken of the peculiar circumstances of the infant economy during that period, in order to prove the necessity of an appropriate plan of conduct in the prevention and treatment of children's diseases: the doctrines of disease will be amply illustrated by clinical observations and cases, drawn from the records and practice of the Dispensary: and physiological opinions of the economy of infants substantiated by facts, deduced from comparative anatomy in regard to the economy of the young of other animals. For particulars apply at the Dispensary.

